# A Treatise on Pathological Anatomy

**Published:** 1831-04-01

**Authors:** 


A TREATISE ON PATHOLOGICAL ANATOMY. By *G. Andral*, M.D. Translated from the French, by *Richard Townsend*, M.D. and *William West*, M.D. In Two Volumes, Octavo, 1831.

IT is needless to observe that the science of pathological anatomy has been cultivated on the Continent, and especially in France, with unprecedented zeal, during the last thirty years—and by none with greater success than by M. Andral. His work is, unquestionably, the best now existing on either side of the Channel, and the translators deserve great praise as well as encouragement for having rendered the original into their native language, by which the labours, and we may conscientiously say the treasures of Andral's work may be diffused through the British profession. The sneers which have too long and too often been cast at the indefatigable and minute unravellings of morbid anatomy on the Continent, are now rapidly subsiding—and the foolish question "*cui bono*," is now hooted out of medical society. If it be true, and we believe it is true, that French physicians pay too much attention to the *effects* of diseases, and too little to the *causes* and to the *treatment* of them, is that any reason why we should run into the opposite error, and despise *one* of the only two means which we possess of investigating the nature, and consequently of successfully treating diseases? To say that it is of no use to ascertain whether inflammation exists in the pleura, or the parenchyma of the lungs, because we must bleed, blister, and purge in both cases, is a melancholy specimen of ignorance as well as laziness:—ignorance, because the seat of inflammation must always make a difference in the mode or the amount of treatment—of laziness, or luke-warmness, because *discrimination* in the diagnosis is always gratifying to the inquiring mind—useful in practice—and highly conducive to the *habit* of accurate observation. If the ascertainment then of the actual and precise seat of a disease bore not, in the smallest degree, on the administration of physic, or the wielding of the lancet, still it would be one of the most profitable of our studies, as sharpening our intellects, clearing away our doubts, and fortifying us against the errors of diagnosis and prognosis, those *opprobria medicorum*, which are far more disgraceful than our failures in cure; because the former *knowledge* (diagnosis) is within our reach, the latter, or methodus medendi, too often beyond all human power. But we shall quit this subject, as the number of ANTI-PATHOLOGISTS, or, as they used vainly and arrogantly to designate themselves, the PURELY PRACTICAL MEN, are now vanishing before the light of science and the ardour of investigation.

There is, we believe, no man now in existence, who has better opportunities, more zeal, or superior talent, for the pursuits of morbid anatomy, than M. Andral. Placed by acclamation at the head of the pathologists of the French school, he may be considered as the chosen organ of that body, and consequently as expressing the present state of the science in that country. His work, accordingly, is unrivalled in the number of original observations which it contains.* But the indefatigable Andral has not confined himself to the dry detail of physical alterations. He has endeavoured to trace up these alterations to their primary shades—to explain the mechanism of their formation—and to ascertain their mutual relation and order of succession. In these investigations he has laboured to restrict the

* "As an instance (say the translators) of the extensive scale on which his pathological investigations have been conducted, I may observe, that he introduces his description of the morbid alterations of the lymphatic vessels, by stating that he examined the thoracic duct in *six hundred* individuals. The number of positive facts collected by one who has enjoyed such extensive opportunities of observation are evidently worth all the theoretical reasonings of the literary compiler, who obtains his knowledge of morbid appearances from books, and compounds his elements of pathology in the study." vii.

influence of inflammation within rational limits, and successfully combats the absurd doctrine, that every alteration of the living structure depends on an exaltation of its vital powers, In short, he has endeavoured to combine the phenomena of diseases with the physical alterations which we detect in the dead body—and thus to afford us certain data for the rational treatment of them. In pursuing this investigation, M. Andral appears to avoid the warping influence of theory. He admits the influence of the solids in the production of morbid phenomena, while he accords considerable importance to the alteration of the fluids. While he admits that local disease is capable of producing constitutional disturbance, he also maintains that those general agents, the blood and the nervous system, may be primarily affected; and that, in this way, general disease may precede the existence of any local affection. M. Andral, in fact, constantly aims at reconciling the jarring interests of adverse doctrines—to select what is of real value from every theory—and thus to profit by all, without wedding himself to any one. In this respect, therefore, the author is a wise eclectic, and the value of a treatise written on such a plan, and by such an author, must be evident.

As to the task of the translators, we conscientiously believe that they have executed it with fidelity, from the comparison of several passages which we have made. With the exception of abbreviating a few cases, the translators have adhered scrupulously to the text of the original. We think we shall best convey an idea of the whole work, by giving a full analysis of some distinct parts—a plan which will prove useful to those who do not possess the work, and, at the same time, offer the greatest temptation to procure it. It is hardly necessary to say that it is in the second volume, containing special pathology, that we must look for a detached subject of analysis, and the first section will furnish us with the first article for that purpose. The first chapter in this section is on the alimentary canal in a healthy state—and the second, on the same part in a state of disease. As we have, in various parts of this journal, presented our readers with accounts of the intestinal canal in states of morbid alteration, especially of its mucous membrane, such as thickening, softening, ulceration, atrophy, perforation, dilatation, contraction, &c. we shall pass over these, and concentrate our attention on the fifth article of the second chapter, entitled—

### State of the Alimentary Canal in the different Cases where its Functions are Deranged.

After having, in former sections, reviewed all the alterations of structure which the alimentary canal presents to the eye of the morbid anatomist, M. Andral remarks justly that, if each of these alterations invariably produced during life a determinate group of well-marked symptoms, we could easily tell what had occurred before death, by the appearances found on dissection. But this is not the case; for, of all parts of the body, the alimentary canal is that in which *identity of lesions* least infers *identity of symptoms*, either local or general. And now our author attempts to answer the following important pathological question:—*the different groups of symptoms to which particular names have been assigned, being given, what is the state of the alimentary canal after death, in each case?*

The greater number of the functional derangements to which the alimentary canal is liable, have, of late, been referred to a state of irritation of the part, indicated in the dead body, 1mo. by various degrees of sanguineous congestion; and, 2do. by various alterations of nutrition and secretion. According to the portion affected, the assemblage of symptoms has got the titles of gastritis, enteritis, colitis, gastro-enteritis, entero-colitis, duodenitis, dothinenteritis, &c. These expressions, observes M. Andral, may be adopted, provided we consider them only as *provisional terms*; the use of which is to suggest to the mind certain alterations, of which *irritation* is the common connecting link, or occasional cause; but which, at the same time, differ widely in their nature and causes, as well as in the functional derangements they produce, and the treatment they require. These derangements are then thrown by M. Andral into four classes—1. Modifications of hunger and thirst—2. Modifications of the phenomena of chymification—dyspepsia—3. Modifications of the phenomena of secretion and excretion—4. Modifications of sensibility.

### 1. Hunger and Thirst.

The sense of hunger may be either inordinately increased (bulimia)—diminished, (anorexia)—or perverted, as in pica. What is the state of the alimentary canal in each of these three cases ?

Bulimia has been attributed to particular lesions, as dilatation of the pyloric orifice—opening of the ductus choledochus into the stomach—superabundance of gastric juice—enlargement of the stomach's capacity—the presence of worms. Although these lesions have more frequently been adduced as the causes of bulimia, from theory than from observation, yet some valuable facts may be gleaned from these hypotheses.

"Thus, in a case of a galley-slave remarkable for his voracious appetite, Vesalius asserts he ascertained that the bile flowed directly into the stomach. Tarara, a celebrated glutton, whose history has been given by M. Percy, had a stomach of enormous size; and Cabrole tells us that, in a person who was continually tormented with insatiable hunger, he found an enormous stomach, terminating in an intestine which, from the place where the pylorus usually exists, to the anus, was only three feet long. However, it may be fairly questioned whether the unusual size of the stomach in great eaters, instead of being the primary cause of hunger, is not rather the effect of the great quantity of food they are in the habit of introducing into it.

*Bulimia* may be produced by a state of irritation of the stomach. It occurs in certain forms of chronic gastritis; but in these cases no sooner is the least quantity of food introduced into the stomach, than total anorexia succeeds to the importunate craving which the patient had previously felt. Many persons labouring under chronic gastritis experience an uneasiness or sensation of dragging in the epigastrium, which they are apt to mistake for hunger.

Bulimia may be completely independent of the state of the stomach, being connected with an habitual or temporary excess of activity in the general nutritive process. Thus, it is frequently observed at the period of puberty, in children when growing rapidly, in convalescents, and lastly, in pregnant women; so that we have here one of the thousand instances of the necessity of seeking the cause of a functional derangement at a distance from the organ whose function is affected." 221.

Anorexia is the most common of all attendants on various morbid alterations of the stomach. Some serious affections of that organ, as mollescence, ulceration, induration of the sub-mucous tissue, give rise to no other symptom but want of appetite! In a few cases of organic disease, where lesion was circumscribed, M. A. found the appetite undiminished. Lastly, in some persons, who had, for a length of time, the greatest aversion to every kind of food, our author was not able to find any appearance in the stomach that could at all account for the phenomenon.

But anorexia, like bulimia, may have its cause in other parts besides the stomach. It occurs in almost all acute and even chronic diseases, evidently from the stomach sympathizing with other structures. Emotions of the mind will take away the appetite more frequently, though less permanently than bodily diseases. Excessive muscular exercise withdraws the power of the stomach and the appetite. In all these various cases, "a modification of the ennervation changes the regular action of the stomach, as it does that of the skin, kidneys, liver, and other organs."

"There are certain states of the system in which most of the actions of the life of relation become suspended, the secretions either cease altogether, or are greatly diminished, and several weeks may elapse without the introduction of any food into the stomach. Such a state occurs naturally every year in hibernating animals. It is probable that, in this singular condition, the process of nutrition of the organs is also suspended, whence the possibility of the creatures doing without food for a very long time; so that here also the modification of the functions of the stomach is only an effect of the modification impressed on more general functions, whose integrity is necessary to the due accomplishment of those of that organ. Thus, by virtue of the *consensus* of all the living parts, the functions of assimilation can only continue perfect so long as the stomach digests properly; and the stomach can digest properly, only so long as there is no derangement in the various functions whose business is to supply or assimilate the nutritive matter to the different tissues." 223.

Pica, or perverted appetite, also may occur as a symptom of chronic irritation of the stomach; but is by no means generally owing to that cause. Hysteria, chlorosis, and pregnancy, more frequently give it birth. In fine, the causes of all the different modifications of hunger may reside in the stomach itself—in the various tissues for which the stomach prepares matter for assimilation—or in the nervous system, or even in the blood. The same may be said of thirst and its modifications. M. Andral is far from believing that the cause of fever is in the stomach because great thirst or even a red tongue may exist. In respect to the red tongue, so triumphantly appealed to by the Broussaians as proof of gastric irritation, M. Andral shrewdly asks—"is it also necessarily the stomach that reddens the conjunctiva, and injects the skin, in those cases?"

### II. Derangements of Chymification.

It is now allowed that dyspepsia, in most of its forms, is owing to gastric *irritation*, and Broussais has done much good by referring it to *inflammation* of the stomach, because that doctrine leads to abstinence, which is as necessary for the cure of irritation as of inflammation.

"Yet it may fairly be questioned, whether every case of dyspepsia necessarily results from gastric irritation. The practice of most French physicians of the present day would apparently authorize our answering this question in the affirmative; but at every period of medical science we find the practice regulated by the prevailing theory, and its efficacy proved by its successful results. It is, however, perfectly well ascertained that some cases of dyspepsia, which had resisted the antiphlogistic treatment, yield readily to other remedies that are any thing but debilitating. And it is equally well ascertained, that in persons who have long suffered from indigestion, the stomach often presents no anatomical lesion sufficient to account for it; and even in those cases where lesions of the stomach are found, it is by no means clear that they have been produced by irritation. If we proceed to investigate the symptoms which attend on this lesion, we find, that there are some which, when combined and strongly marked, appear to be so little the result of irritation, that they are advantgeously treated, nay, speedily removed, by means that are themselves irritating." 226.

It is rather difficult to ascertain precisely what M. Andral means by *irritation*, and whether he makes any essential difference between that and *inflammation*. Speaking, in the next section, of "dyspepsia from gastric *irritation*, acute or chronic," he observes that *this* is, beyond all contradiction, "the kind of dyspepsia most commonly met with"—and no doubt it is—but very frequently an irritation totally unaccompanied by inflammation. Contrary to M. Broussais, our author admits the existence of atonic dyspepsia, or weakness of stomach.

"This is observed to occur, 1. under the influence of causes altogether unknown; and 2. subsequently to certain appreciable modifications of the system. Thus, it sometimes happens that the stomach, after having been more or less irritated, falls into the opposite state, and becomes affected with genuine atony. The same happens in some cases of convalescence. This state of atony likewise occurs in persons exhausted by excess in venery, and, still more, by onanism. It would be a serious mistake in such cases to imagine that dyspepsia is always the result of gastric irritation; for that would lead to the employment of remedies which frequently aggravate the complaint. Under such circumstances, I have seen dyspeptic affections yielding rapidly to a tonic regimen, which could not have failed to exasperate the irritation of the stomach, if such there had been. Why may there not be weakness of the stomach as well as of the muscles in those cases? Dyspepsia from weakness of the stomach is generally attended with a complete absence of thirst, and great paleness of the tongue. The patient experiences, after taking food, a sensation of weight in the epigastrium, and frequently a tension in the same region. The use of gum water sometimes produces these symptoms, as do also the infusions of linden and chamomile, though in a less degree. This particular state of the stomach may render the pulse somewhat more frequent; but as we have already seen more than once, frequency of the pulse is not necessarily connected with a state of irritation." 228.

The third genus of dyspepsia adverted to by M. Andral, is considered as dependent on "alteration of the follicular secretion of the stomach." This has long been distinguished by the French physicians under the vague term "EMBARRAS GASTRIQUE," the nature of which is far from being well known. It does not yield to blood-letting—and but slowly to abstinence; —"while it readily gives way to emetics and purgatives." That it is often connected with gastric irritation, he admits—and then the symptoms are exasperated by tartar emetic. But he is far from agreeing with the Broussaians that, in every instance, it is a form of *gastritis.* He presumes that it depends on an altered secretion of the gastric glands; and he cannot see why this "should be considered as necessarily the result of *irritation*, unless we choose to allow that the mucous coating which covers the tongue, necessarily indicates glossitis." This passage shews that M. Andral makes no distinction between irritation and inflammation. This is a sad oversight in our Parisian pathologist!

The next, or FOURTH GENUS of dyspepsia, is attributed to "MODIFICATION of the NERVOUS INFLUENCE."

"We know that the process of chymification may be modified by deranging the innervation of the stomach either by administering opium, cutting the pneumo-gastric nerves, or producing a strong mental emotion. In these various cases, the digestive powers are not so much increased or diminished, as perverted from their natural condition. Certain cases of dyspepsia seem likewise to result from this modification of the nervous influence. Against these, antiphlogistics and tonics are equally unsuccessful, and the reason is evident; for the indication of cure is neither to weaken nor to excite the stomach, but to modify its action." 230.

This indication leaves us pretty much in the dark, as to remedial processes. We are neither to lower nor increase the tone of the stomach:—but to *modify* its action. We suppose this must be to *change* its action, in the same way that the *modes* are changed in Paris. The plain indication is, to search for the cause, and endeavour to remove it—though that, unfortunately, is not often in our power. Suppose it is a moral cause, as domestic affliction, wounded feelings, or pecuniary losses. The nervous energy of the stomach is weakened—or, as our author would term it, modified, perverted. Well. Neither tonics nor antiphlogistics will do any good. What are we to do? Should we not simplify and reduce the diet, so as to give the organ as little to do as is consistent with the preservation of life, and render the operation of digestion as easy as possible? This will be the *practical* rule, whatever pathologists may say—and the one which will tend to allay the sufferings of the patient, and enhance the reputation of the practitioner.

### Derangements of Secretion and Excretion.

Under this head our author considers vomiting, diarrhœa, cholera. We find, after death, all kinds of anatomical changes, without any previous vomiting or purging—but yet every one of these lesions occasionally gives rise to the symptoms in question.

"In the first place, we find vomiting produced by all possible degrees of irritation of the gastric mucous membrane, which cause the patient to throw up either his food and drink, or else *the bile that had previously been attracted by the irritation into the stomach;* in other cases the matter vomited consists of blood exuded by the irritated mucous membrane, or of mucus secreted in superabundant quantity; which last, by accumulating, becomes a kind of foreign body, and produces by its presence a secondary irritation more considerable than that to which it owed its existence." 231.

We very much doubt the propriety, or even the truth of the assertion that an irritation in the stomach will *attract* bile into that organ. Is it not much more likely that the bile is regurgitated from the duodenum into the stomach, during the action of vomiting, than that it should be attracted there by either inflammation or irritation? Even when bile is found in the stomach, without previous vomiting, it is from an inverted action of the duodenum rather than from any suction power in the stomach.

Poisons which irritate and inflame the stomach, almost invariably produce vomiting; " and yet, in the different varieties of fever, which M. Broussais considers as so many cases of acute gastro-enteritis, this symptom is observed but very seldom." Mechanical obstructions of the pylorus, or any other part of the tube, will produce sickness and vomiting. But it is curious that irritation or inflammation of the *serous* membrane of the stomach or bowels, is more apt to cause sickness, than the same diseases in the mucous tissues. " Indeed, nothing is more common than to find it (the mucous membrane) white and healthy in persons dying of acute peritonitis, who had been continually vomiting to the last moment of their lives."

The cause of vomiting is not always in the stomach itself, but must be referred to the nervous centres. Magendie's experiments shew that, when the stomach of an animal is removed, and a bladder substituted, tartar emetic injected into the veins will cause attempts to vomit, and, if the bladder be properly placed, actual vomiting. The sympathetic causes of vomiting are very numerous, as the sight of disgusting objects, sea-sickness, irritation of the fauces, pregnancy, nephritis, diseases of the brain, &c. These kinds of vomiting leave no traces after death in the stomach itself. After these considerations, and in the actual state of our knowledge, M. Andral thinks that " the expression *nervous vomiting* deserves to be retained, as signifying a real morbid state, in which the gastric symptoms that occur have their source, not in the stomach itself, but in the brain, whose texture or action is decidedly modified."

" One of the most striking instances of these nervous vomitings is that recorded by M. Louyer Villermay : the subject was a woman, who, in consequence of a disappointment in love, was attacked alternately with globus hystericus, dyspnœa, and palpitation ; she uttered involuntary cries, and at last was seized with vomitings, which were treated in vain by the antiphlogistic method *(diet, emollient drinks, leeching.)* She was at last cured by the *vinum absinthii,* and the first food she digested was hard-boiled eggs and salad." 234.

We may take this opportunity of alluding to the fashionable ridicule which is now thrown upon the terms *nervous* and *sympathetic* disorders, by a class of medical gentlemen, who are more conversant with the dead than with the living body—with the dissecting-room than with the sick-room. Yet nothing can be more proper than the term *nervous disorder* in a great proportion of human ailments. Nine-tenths of our diseases—even those which leave in the dead body most palpable traces of their existence, are purely *nervous* affections at first—that is, troubles of function, produced solely through the instrumentality of the nerves. Then we must recollect that the brain and great nervous centres are perpetually getting deranged in their functions, from the ten thousand insalutary agents that surround us, and where no organic change is produced perhaps at all—or, if produced, it is by long continuance of the functional disorder, and is then an effect, not a cause of the phenomena of the primary complaint. The testimony of a man so much devoted to pathology as M. Andral is, must also have some weight with the anti-neurologists.

Even the various intestinal fluxes, as dysentery, diarrhœa, and lientery, are not invariably dependent on any peculiar condition of the alimentary canal.

" It is true that, in the greater number of cases, we find in the intestines of persons who have had any kind of looseness, various alterations, of which the primary cause and common connecting link is a process of irritation, past or present. These alterations exist most frequently in the great intestine, of which they sometimes occupy the whole, and sometimes only a part. There are some cases where the cæcum, and others, where the rectum alone is diseased. The small intestine may continue sound through its whole extent; and one is sometimes struck with the abruptness with which the morbid state commences immediately below the ileo-cæcal valve. In other cases, on the contrary, there is no sign of any lesion to be found in the great intestine, and the end of the ileum is the only part diseased. The most common case is that in which the inferior extremity of the ileum and a more or less extensive portion of the great intestine are simultaneously affected.

These alterations, which have been all described already, from simple injection to ulceration and perfect softening, bear no constant proportion, either to the duration, or the symptoms of the disease. Thus, in several persons who have had a diarrhœa for the same space of time, from eighteen months to two years, for instance, and in whom the disorder has been attended with the same series of phenomena, local and general, there may be found in one a simple red or brown hyperæmia, without any other alteration; in another, a red softening of the mucous membrane; in a third, a white softening of the same; in a fourth, induration with various shades of colouring; in a fifth, an unusual development of the follicles, without any other lesion; in a sixth, ulcers of various sizes, and in various numbers; and, lastly, in a seventh, some one or other of the above-mentioned alterations of the mucous membrane, together with various morbid states of the subjacent tunics, such as thickening, serous infiltration, &c. As to the symptoms, there is not one that announces with certainty that the intestine is affected with one particular lesion rather than another. Thus we find the same alterations where there has been a serous and bilious diarrhœa, as where there has been dysentery. There is not unfrequently complete absence of fever and of pain in those cases where the intestine is crowded with ulcers, and the mucous membrane is greatly thickened, and of a red, brown, or black colour.

Does dissection discover, in the bodies of all who die while affected with intestinal flux, some one of the alterations just mentioned? This has been asserted, but the assertion is not supported by facts. The researches of modern anatomists have clearly proved, that there are certain cases in which dissection cannot discover any appreciable alteration either in the colour, thickness, or consistence of the intestinal parietes, or the appearance of the follicles, &c. In some of these cases the diarrhœa commenced only a few days before death, and in others had been of a long standing. Here, then, is another case where the appearance of the alimentary canal after death does not lead to any certain knowledge of the functional derangements with which it had been affected during life." 237.

The liver sometimes disgorges such quantities of acrid bile, as to cause diarrhœa, without any primary affection of the intestines themselves. In cholera morbus, our author acknowledges that morbid anatomy throws no light on the nature or cause of the malady.

The fourth class is headed "MODIFICATION OF THE SENSIBILITY." Severe pain, M. Andral observes, seldom accompanies any of the various alterations of texture, in the gastro-intestinal mucous membrane. It may be injected, softened, thickened, or deeply-ulcerated, without the patient's complaining of any uneasy sensation—or, at the most, of more than some griping pains on going to stool. This is the case, not only in the chronic, but also in the acute state of these lesions. We may, in general, press the abdomen in all directions, in persons labouring under severe fever, and whose intestines are considerably ulcerated. It is only when the ulcers extend in depth, so that their bottom is composed solely of peritoneum, that severe pain is felt.

"While the most serious alterations in the texture of the gastro-intestinal mucous membrane produce little or no pain, there are other cases in which the alimentary canal becomes the seat of very severe pain without its texture being at all altered; as in the disease known by the name of colica pictonum, or saturnine colic. Some writers have asserted that in cases of this affection they found, on dissection, the caliber of the intestines remarkably diminished; others, again, that the symptoms arose from introsusception; and lastly, others, that in persons who have died of the disease, the intestine was found more or less injected, and that, consequently, this disease was a species of enteritis. It is very possible that there may have been found, in the intestines of persons who died of saturnine colic, diminution of caliber, introsusceptions, or various degrees of redness; but all these lesions seem to me to be merely accidental, since, on the one hand, they may occur without producing the symptoms of colica pictonum, and, on the other, they are not invariably found in persons who have died of the disease. I have related elsewhere* in detail several of these cases in which I was not able to discover any appreciable lesion in the alimentary canal. M. Louis has related others of the same description. Neither could I, in a single case, discover any lesion of the nervous centres, and yet there had been remarkable paralysis in most of them." 240.

During the campaigns in Spain, ample opportunities were found to examine the bodies of people who had died of the Madrid colic, a disease very similar to that from lead. Dr. Pascal has published an account of six dissections of this kind, made by himself; and the result was, that in the colic of Madrid, the lesions of the alimentary canal are neither serious nor constant. In three of the cases out of six, there was slight redness of the intestine. The spinal cord, in all these cases, was strongly injected.

### State of the Intestinal Canal in Fever.

Fever must have been one of the first diseases that attracted the attention of mankind. The local affections that are often found to be complicated with fever were, first, considered as *effects* of the disease—and more recently as *causes* of it. The stomach and the brain have been selected, as all our readers know, for the thrones and seats of fevers, by large parties in this and other countries—doctrines that are not quite so new as their modern renovators or inventors appear to think. Bonetus long ago observed that—"Anatomia eorum qui febre maligna extincti sunt, docet *ventriculum cum intestinis* inflammari;" while Bartholinus says—"in *omni* febre acuta imminet ventriculi inflammatio." Sydenham informs us that the intestines become ulcerated inc ontinued fevers; but as he has not given us a single dissection throughout his works, the pathological doctrines of that eminent physician do not stand on very high ground. The following extract will shew M. Andral's opinions respecting fever.

"To seek, in the alteration of one or more parts of the body, the seat and the cause of the fevers called essential; to consider them as merely the symptoms of a local affection more or less manifest; and to direct the treatment

* Clinique Medicale.

against that affection, and not against the fever itself, which is merely an effect, are parts of the doctrine of M. Broussais which have already produced a most extensive and important revolution in medical science. These tenets, however, form but a part of his doctrine: he has also attempted to specify the lesion which alone, in his opinion, can give rise to the various symptoms that characterize essential fevers, and he maintains that all such fevers are the result of gastro-intestinal irritation. Here he has failed; and while I agree with him that every kind of fever may be attributed to a local source, I think that the localization should not be at all so confined as he has made it. On this head, the following propositions appear to me to give a pretty accurate idea of the present state of our knowledge with respect to the seat and nature of fever.

There is no essential fever, so called, which may not be referred to the alteration either of some solid or of the blood, as its cause. These essential fevers, then, are not general diseases, inasmuch as they have always a local origin; but they may be considered general in this sense, that, being sometimes seated in the nervous centres, or in the blood, they produce a disease all over the body, wherever the blood and nerves are distributed, or, in other words, a general disease.

With respect to their local origin, fevers may be divided into three classes; the first consisting of such as arise from a primary alteration of the nervous centres; the second, of those arising from the lesion of a solid; and the third, of those arising from a modification of the blood.

The fevers belonging to the first class are distinguished by that assemblage of symptoms which M. Pinel has assigned to adynamic and ataxic fever, especially the latter. The former, however, more frequently occurs when the disorder of the nervous centres is subsequent to an affection of the alimentary canal, or some other part. Although the symptoms of those fevers plainly point out their seat during life, dissection sometimes discovers no more alteration in the nervous centres than in the other organs. In my opinion, this absence of alteration does not affect the determination of the seat of the disease; that having been too clearly indicated by the derangement of the functions to be mistaken. Will any one assert that epilepsy or tetanus is a general disease, because he cannot discover any lesion in the bodies of persons who have died of either?

Fevers of the second class depend on the primary alteration of a solid; this alteration may be, 1. in the quantity of blood it receives; 2. in its texture; or, 3. in its functions only. The solid primarily affected is certainly in a large proportion of cases the alimentary canal, but it may also be the skin, lungs, liver, kidneys, heart, vessels, uterus, ear, prostate, &c. I have mentioned these different parts, because I am in possession of facts that prove that the alteration of any one of them, without any concomitant lesion of the alimentary canal, is capable of giving rise to the symptoms that constitute the different essential fevers. The tongue, in some of those cases I allude to, was red, in others thickly coated, and in others dry and black, and yet on dissection there was no lesion discovered either in the stomach or intestines.

In these various cases, we are far from being always able to establish a constant proportion between the intensity of the lesion, and the nature of the symptoms that constitute the fever; as they depend much less on the severity of the local lesion, than on the state of the innervation and sanguification that happens to exist in the individual in whom the lesion occurs. In fact, on this circumstance it depends whether the slightest lesion may not occasion a most serious ataxic or adynamic fever; and a much more severe one produce only inconsiderable symptoms.

Adynamic fever, which is so often occasioned by these various lesions, is a complex term comprehending several morbid states differing greatly with respect to their nature and proper treatment. It is often merely the result of a considerable oppression of the vital powers, produced by a local lesion. But, frequently also, when the lesion occurs, the nervous centres have scarcely re-acted before they fall into a real state of collapse, and in this case the adynamic condition is genuine; the nervous influence by which every organ lives has really lost its energy; and throughout the whole body life is less active, and the resistence to the return of the organized being to the dominion of the laws of physics is less strong; we have here a genuine adynamic state produced by an increase of vitality in some part of the body. In such cases it may happen that along with the signs of prostration of strength we observe some symptoms of cerebral excitement; but such symptoms are only factitious, as the adynamic state is in other cases. We must therefore beware of supposing that subsultus tendinum, convulsions, and delirium, are always signs of increase of cerebral life: they are so far from being necessarily so, that they sometimes occur after abundant hæmorrhages.

Lastly, the third class of fevers appears to be more particularly connected with alterations of the blood. On this head, I can but refer to what I have already said in the first volume, when treating of the diseases that may arise from that source. I shall content myself at present with repeating that the fever termed *inflammatory*, seems to me often to arise from no other source than the blood's being too rich in fibrine; in like manner an impoverished state of the blood, whether accidental or natural, is often connected with mucous fevers, and with those characterized by a sudden sinking of the vital powers: the blood's not being sufficiently depurated is certainly the cause of the fever termed *urinous*, and probably that of certain bilious fevers also; lastly, the source and primary seat of typhus fevers, properly so called, is proved to be in the blood, inasmuch as they are caused by the introduction of deleterious substances, such as animal or vegetable effluvia, into that fluid." 252.

The above is a moderate *eclectic* doctrine, and probably more near to the truth than any which has recently been propounded. The abrupt denial of the existence of such a thing as *idiopathic* or general fever, is soon qualified by the admission that the fever is sometimes as *general* as the distribution of the *blood and the nerves*—which is latitude enough for any man.

The last section in this division of the work, treats of "the state of the alimentary canal in the diseases of various organs." It has long been known that the alimentary canal exercises an immense influence on other organs of the body, and also that it is readily influenced in its turn, by the disorders of those other organs or parts. M. Andral calculates that, in eight out of ten cases of acute diseases of other organs, the alimentary canal will be found to suffer either in function or structure. In *function*, we would say that it is hardly possible for the alimentary canal to escape, when any acute inflammation exists in any part of the body.

In chronic diseases the digestive apparatus is almost certain to suffer. This derangement is sometimes permanent, and becomes chronic like the disease with which it is complicated—in other cases it is periodical.

"The gastro-intestinal irritation that occurs thus intermittingly may produce no effect on the primary chronic affection; but the reverse frequently happens. There are cases, for instance, in which, whenever the irritation of the digestive passages re-appears, the chronic affection is exasperated, and has a tendency to return to the acute state; there are others in which, on the same occasion, the old affection, so far from being exasperated, is so much amended that its symptoms become much less apparent. Lastly, in many cases, whether the primary chronic affection be unaltered, aggravated, or relieved, the gastro-intestinal irritation produces another effect: it acts on the innervation, and produces in the patient exhausted by long disease that assemblage of symptoms that characterizes the adynamic fever of Pinel. Under such circumstances, a slight irritation of the alimentary canal is sufficient to produce a great and sudden prostration of strength. Many persons affected with a chronic disease that has long been undermining the constitution, sink thus under an adynamic fever arising from a trifling congestion in the alimentary canal. The danger of such a hyperæmia is not so much in proportion to its intensity, as to the diathesis of the patient at the time it occurs." 253.

Chronic irritation in the mucous membrane of the chest seldom fails to affect this same membrane below the diaphragm, and vice versâ. The alimentary canal is so generally diseased in tubercular bronchitis, that such disease becomes as it were, one of the elements of phthisis, and almost makes a constituent part of it.

In about four fifths of the pthisical patients that die at an advanced period of the complaint, the intestines are found greatly diseased—and that by ulceration, generally about the end of the small intestine, and the cæcum. These ulcerations are very variable in number, size, and form; and are commonly formed without pain, producing merely diarrhœa. There are, however, alternations of diarrhœa and constipation, at first, generally ending in confirmed diarrhœa. The stomach is often affected in phthisis, but in a different manner—not by ulceration, but by softening of the mucous membrane, or attenuation of the same.

"What is the cause of the dyspepsia or of the pains in the stomach experienced by many women affected with leucorrhœa? Do they suffer merely from simple gastric irritation? Is the morbid modification that takes place in their stomach, of the same nature as that which the utero-vaginal mucous membrane has undergone? If the alteration of secretion that takes place in the latter is often removed only by treatment that is any thing but antiphlogistic, are we to employ the same treatment to relieve the stomach? There is still some light wanting on this subject: however, as it is always advantageous to the cause of science to publish facts for the accuracy of which we can answer, I shall state briefly the following case. A woman was admitted at *La Charité* with a leucorrhœa of long standing, difficulty of digestion, and a pain in the region of the stomach. I prescribed leeches to the epigastrium, without effect; and opiates were found equally unsuccessful. I then tried pills of extract of bark and iron filings, whereupon the pain in the epigastrium diminished, and digestion was restored." 256.

Cutaneous affections, our author observes, are accompanied, as frequently as those of the various mucous membranes, by a diseased state of the *primæ viæ*. Thus, in persons who die of extensive burns, the alimentary canal is generally found of an intensely red colour. In many cases of erysipelas, the stomach is affected at the same time. At the commencement of the febrile exanthemata, there is almost always a congestion of one of the mucous membranes. In scarlatina, it is the mucous membrane of the pharynx—in measles, the air-passages—in small-pox, the stomach. Before the eruption of this last disease, the gastric lining membrane generally presents only a slight irritation, which diminishes rather than increases when once the eruption appears. In some cases, however, this precursory irritation is very severe, and continues after the eruption comes out.

"The gastro-intestinal irritation, though slight at the commencement, may become much more intense during the course of the disease; and in many cases where the small-pox is termed putrid, malignant, adynamic, &c., it is merely complicated with a more or less severe gastro-enteritis, which, on the one hand, has modified the eruption, and on the other, has reacted on the nervous centres. When the disease proves fatal, there are found in the alimentary canal the various alterations already described, from hyperæmia to ulceration. Are there also found in it pustules similar to those that cover the skin? Since they are observed very distinctly on the mucous membrane of the cheeks, there is no reason why they should not also occur on the portion of the membrane situated lower down. But, though in theory the existence of variolous pustules in the intestine is admissible, they have never yet been seen there. The mucous follicles, more developed than ordinary, have often been taken for them; such a mistake is particularly easy in children, in whom these follicles are often very large." 258.

The chronic exanthemata are attended with a state of irritation in the digestive organs, much less frequently than has been asserted—at least this is the opinion of M. Andral. He instances as proof, the strong medicines which M. Biett so happily, and so successfully employs in the St. Louis Hospital. But there are other states than that of irritation or inflammation in the stomach and bowels. The functions of these parts may be greatly disordered in other ways, and may be much bettered by active medicines administered internally.

The following passage concludes this division of the work.

"One of the most important services which M. Broussais has rendered to medicine, was the proving that, in many cases where, from the functional derangements, one would suppose the nervous centres were alone affected, the origin of the disease is really to be found in an irritation of the alimentary canal. This is true of all ages, particularly of infancy. In children it frequently happens that, after coma, convulsions, &c. there is no morbid appearance to be discovered in the brain, while the alimentary canal is evidently diseased.

A great many functional derangements of the nervous centres have been referred to irritation of the digestive tube, because the intestines have frequently been found diseased in cases where such derangements had existed. Thus, it has been asserted that tetanus, epilepsy, chorea, apoplexy, and alienation of mind, result from gastro-enteritis. In my opinion, there is not a nervous disorder that may not be developed in consequence of an irritation of the alimentary canal, as well as of any other part; but then a previous disposition to it is requisite.

On the other hand, the nervous centres, when primarily affected, often exercise as great an influence over the alimentary canal as it does over them; but the influence is not always of the same kind: sometimes, for instance, irritation of the brain produces in the stomach a hyperæmia demonstrable by dissection, and sometimes an excitement indicated by the derangement of its functions, but not discoverable by the anatomist. Thus it often happens, as we have already remarked, that we cannot discover any lesion in the stomach of persons who have been affected with copious vomitings in consequence of acute hydrocephalus. Lastly, in other cases, diseases of the brain affect the alimentary canal with a kind of torpor, so that emetics and purgatives do not produce any effect." 261.

From this analysis of a single division of M. Andral's Pathology, our readers will be convinced of its value, and great practical utility. In some succeeding articles, we shall offer further proofs of this favourable opinion.

